# Differences in gross appearance and histopathology of the outer membrane of the subdural hematoma envelope over time: A respective case series and literature review

**DOI:** 10.1097/MD.0000000000034257

**Published:** 2023-07-21

**Authors:** Han Seung Ryu, Sung Sun Kim, Woo Jun Hong, Tae-Sun Kim, Sung-Pil Joo

**Affiliations:** a Department of Neurosurgery, Chonnam National University Hospital and Medical School, Gwangju, Republic of Korea; b Department of Pathology, Chonnam National University Hospital, Gwangju, Republic of Korea.

**Keywords:** elderly patient, head trauma, histopathologic study, subdural hematoma

## Abstract

Chronic subdural hematoma (CSDH) is a common disorder among elderly males. The most common theory of its cause is a minor brain injury resulting in the rupture of a bridging vein. The outer membrane of subdural hematoma (SDH) evolves like cutaneous wound healing with different phases. This report aims to use a surgical microscope and an electron microscope to show the pathophysiological differences in the temporal flow of the outer membrane of SDH.

**Patient concerns:**

This study retrospectively reviewed the cases of 6 patients who underwent craniotomy from 2016 to 2021 at the single center of Chonnam National University Hospital.

**Diagnoses:**

These patients had a history of intracranial hematoma (ICH) at the surgical site on brain computed tomography (CT) before craniotomy. This study aimed to observe the morphological changes over time in the outer membrane of SDH and analyzed them through macroscopic and pathological findings.

**Interventions and Outcomes:**

The outer membrane of SDH was confirmed in all six patients who underwent surgery, and macroscopic analysis was performed using an operating microscope. Three patients underwent pathological analysis through histological examination, and through this, the difference according to ICH occurrence and detection time was analyzed.

**Lessons:**

This study suggests that the outer membrane of SDH contains inflammatory and collagen cells in the early stages and thickens over time. This healing response is similar to cutaneous wound healing.

## 1. Introduction

Chronic subdural hematoma (CSDH), a cystic uncoagulated hematoma with adventitia and intima in the subdural space, is one of the most common disorders encountered by neurosurgeons. It has a prevalence of 1 to 2 cases per 100,000 people, and the majority are elderly males. In developed countries, the incidence rate is increasing due to the extended life expectancy.^[1,2]^

The most common cause of CSDH is a minor inertial brain injury triggering brain movement within the skull, resulting in the rupture of a bridging vein that traverses the cell layers at the dural border.^[3]^ However, approximately 30% to 50% of CSDH patients have no history of trauma, and risk factors, such as alcohol abuse, seizures, clotting disorders, cerebrospinal fluid shunts, and anticoagulant therapies, are known to be involved in the development of CSDH.^[1,4,5]^

The evolution of CSDH appears to parallel cutaneous wound healing, with phases of clotting of any extravasated blood, inflammation, proliferation, formation of granulation tissue, and remodeling and healing with fibrosis. Yamashima et al^[6]^ published a pathological report on a CSDH 30 years ago. Since then, advances in science and imaging technology have made many advances in the pathophysiologic of CSDH. This report intends to show the pathophysiological differences in the temporal flow of the outer membrane of CSDH through surgical and electron microscopy.

## 2. Materials and methods

We retrospectively reviewed cases of patients who underwent craniotomy from 2016 to 2021 at a single center at Chonnam National University Hospital. A total of 6 patients with previous intracranial hematoma (ICH) history at the surgical site indicated by brain computed tomography (CT) performed before craniotomy were reviewed. Among them, 3 patients were analyzed via a surgical microscope, and the remaining 3 patients underwent a biopsy of the outer membrane of the subdural hematoma (SDH) to analyze gross and pathological findings. The 6 patients had different timing of intracranial hemorrhage before craniotomy. These characteristics were suitable for our study to observe the morphological changes of the outer membrane of CSDH over time (Table 1). This study was conducted following the tenets of the Declaration of Helsinki (6th Amendment, 2008) and satisfied all the requirements for patient anonymity. Informed oral consent was obtained from each patient and guardian for inclusion in this research study.

**Table 1:** Clinical characteristics and histopathological features of chronic subdural hematoma membrane.

No.	Age	Sex	Present problem	Previous ICH Hx.	Duration between ICH Hx to craniotomy	Histopathologic exam	Histopathologic finding		
							LCA	CD 34	Masson trichrome stain
1	60	F	SAH	+	1 mo	None	-	-	-
2	61	M	Unruptured cerebral an.	+	3 mo	None	-	-	-
3	53	M	Unruptured cerebral an.	+	1 yr	None	-	-	-
4	64	F	Subacute SDH	+	2 wk	Done	+++	+++	Not layered
5	72	F	Unruptured cerebral an.	+	8 mo	Done	++	++	Thin layer
6	71	F	Intracerebral hemorrhage	+	4 yr	Done	+	+	Thick layer

ICH = intracranial hemorrhage, LCA = leukocyte common antigen, SAH = subarachnoid hemorrhage, SDH = subdural hemorrhage.

### 2.1. Illustrative cases

#### 2.1.1. Cases for using surgical microscope.

Three patients had a history of neurosurgical treatment due to intracranial hemorrhage. A membrane thinner than dura was observed in the subdural space through an operating microscope when a craniotomy was performed after a certain period from the time of hemorrhage discovery.

**Figure 1.**
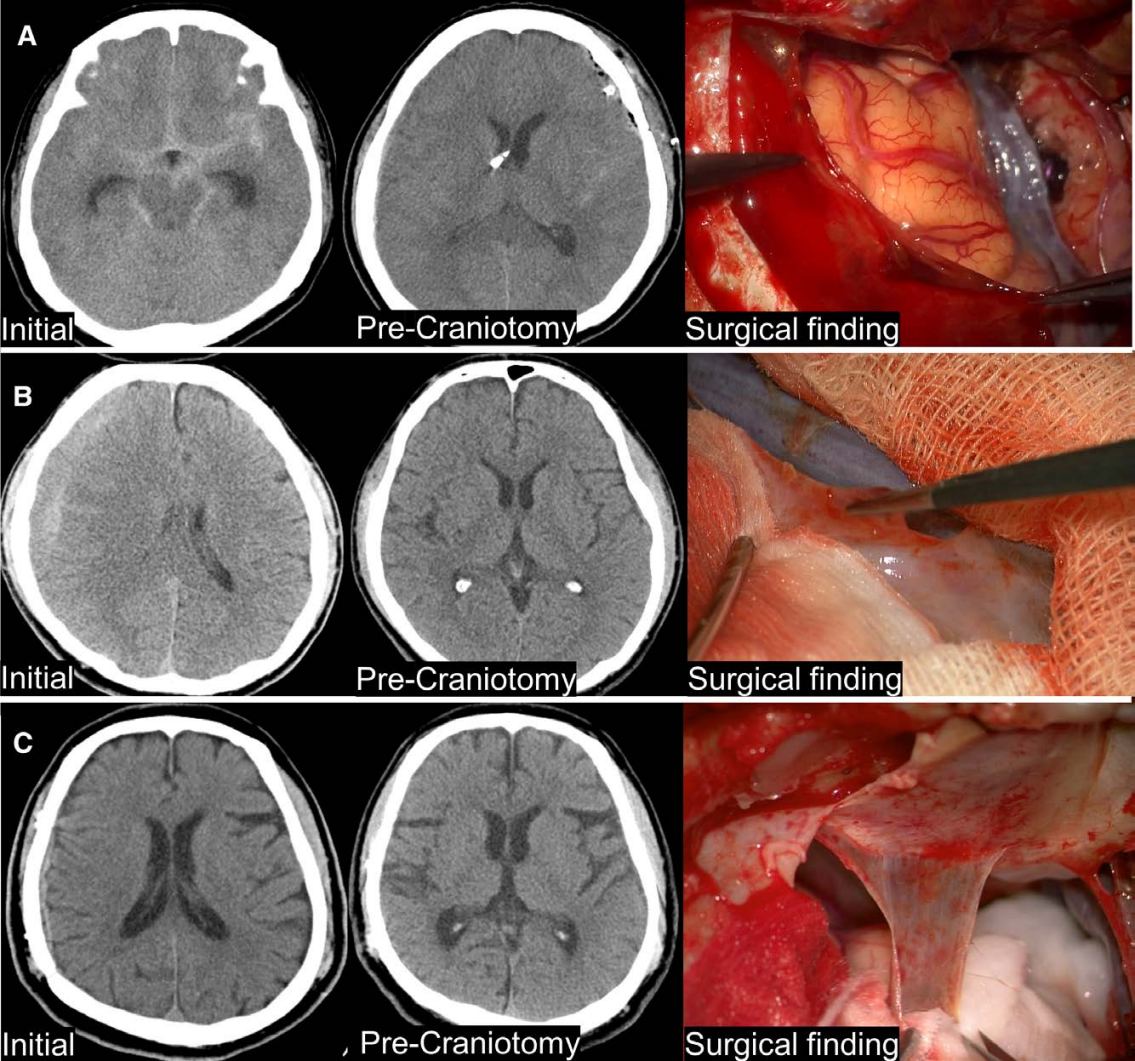
Three cases and gross findings of SDH membrane using surgical microscope. Brain CT showing the history of intracranial hematoma (ICH) in Patients 1, 2, and 3, brain CT with preoperative ICH resolution, and pseudomembrane seen during craniotomy were sequentially listed. You can see the difference in gross findings through the surgical microscope of the pseudomembrane. CT = computed tomography, SDH = subdural hematoma.

One patient was a 60-year-old female with SAH due to a ruptured aneurysm. Upon examination, both middle cerebral artery bifurcation (MCAB) aneurysms were found, so an emergency clipping operation was performed for the left ruptured MCAB aneurysm. After 1 month, she underwent a craniotomy for the contralateral aneurysm clip (Fig. 1A). Another 61-year-old male patient presented with CSDH of right-side convexity, and trephination was performed on the affected area. After 3 months, an unruptured right MCAB aneurysm was discovered incidentally during the outpatient follow-up, and the patient was subjected to craniotomy for clipping (Fig. 1B). A 53-year-old male patient underwent conservative treatment for subdural hemorrhage in the right convexity after head trauma. One year after head trauma, the patient underwent a clipping operation on the anterior communicating artery aneurysm, which was incidentally discovered during outpatient follow-up (Fig. 1C).

In all 3 patients, the SDH at the site of craniotomy was absorbed entirely on the brain CT just before the operation. Although it was not visible on the CT scan, there was a translucent membrane thinner than the dura mater in the subdural space during the operation. These membranes differed in thickness and transparency when observed under a surgical microscope. The larger the difference between the first hematoma and the time of craniotomy, the thicker and less transparent membrane could be observed.

#### 2.1.2. Cases using surgical & electron microscopes.

Three other patients underwent craniotomy 4 weeks, 8 months, and 4 years after acute SDH due to head trauma, respectively. According to the surgical findings of these patients, thin translucent membranes of different thicknesses were observed in the subdural space under the operating microscope. These membranes were collected, and pathological studies were performed with an electron microscope.

**Figure 2.**
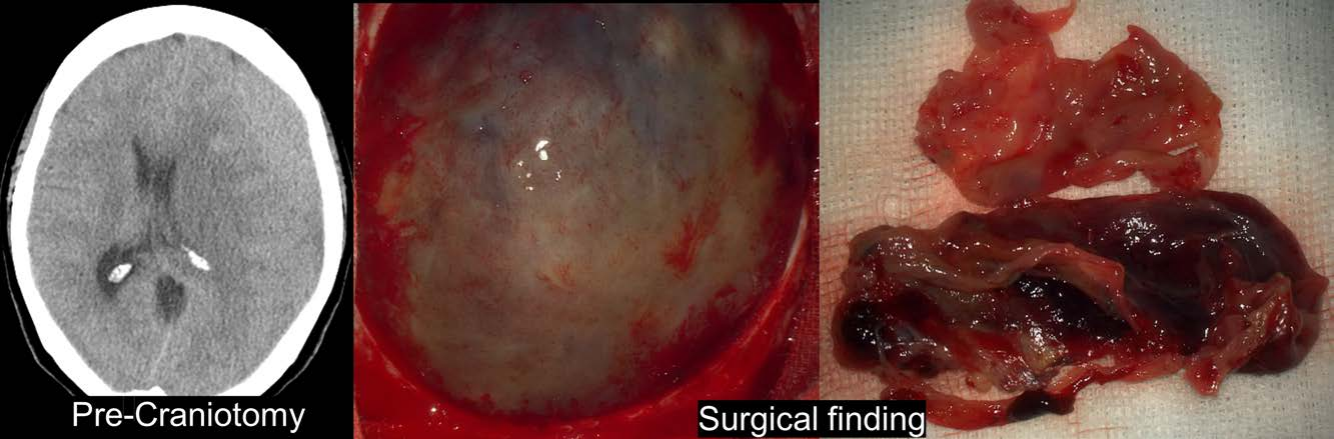
Gross findings of SDH membrane for patient 4. Brain CT showing the history of intracranial hematoma (ICH) in patient 4 and pseudomembrane seen during craniotomy were sequentially listed. Visual findings can be confirmed through an operating microscope of the pseudomembrane. CT = computed tomography, SDH = subdural hematoma.

**Figure 3.**
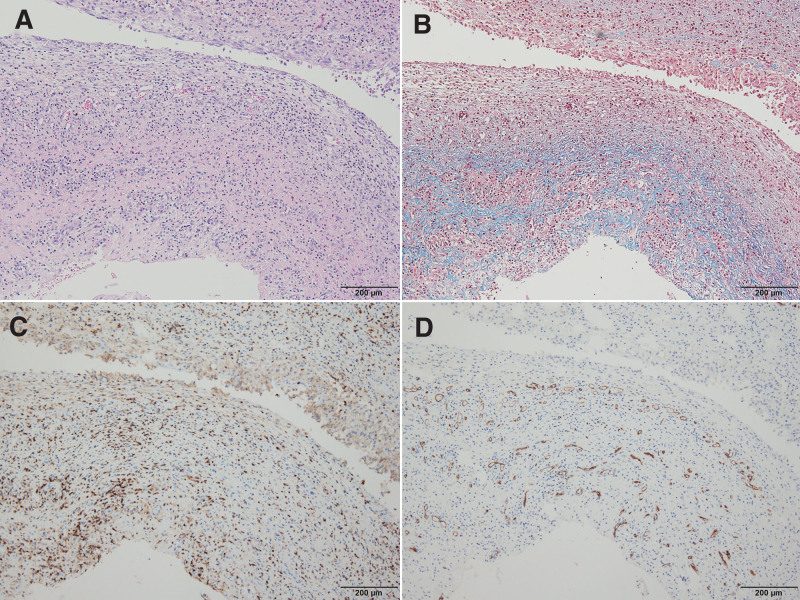
Histopathologic findings of SDH membrane for patient 4. (A) There was thick membranous structure, which is composed of Compact surface layer, intermediate layer including blood vessels, numerous inflammatory cells and fibrocollagenous tissue, and the arachnoid Surface layer. (B) Masson trichrome stain revealed moderate amount of fibrous tissue in the intermediate layer. (C&D) Immunohistochemical stain for LCA (leukocyte common antigen) and CD34 showed numerous inflammatory cells and many thin-walled vessels within the intermediate layer (A. Hematoxylin & eosin stain, original magnification, X200, B. Masson trichrome stain, X100, C. LCA immunohistochemical stain, X100, D. CD34 immunostain, X100). SDH = subdural hematoma.

One patient was a 64-year-old female who underwent trephination due to chronic subdural hemorrhage and had rebleeding 2 weeks after surgery. A craniotomy was performed to remove the hematoma. The surgical findings showed a very thick translucent membrane, and a hematoma tightly attached to this membrane was observed (Fig. 2). In the histopathological studies, hematoxylin and eosin (H&E) staining revealed a thick membrane structure consisting of a dense superficial layer, an intermediate layer containing blood vessels, numerous inflammatory cells, fibrous collagen tissue, and a superficial arachnoid membrane (Fig. 3A&B). Masson trichrome staining showed a moderate amount of fibrous tissue in the middle layer, and immunohistochemical staining for leukocyte common antigen (LCA) and CD34 showed many inflammatory cells and thin-walled blood vessels in the intermediate layer (Fig. 3C&3D).

**Figure 4.**
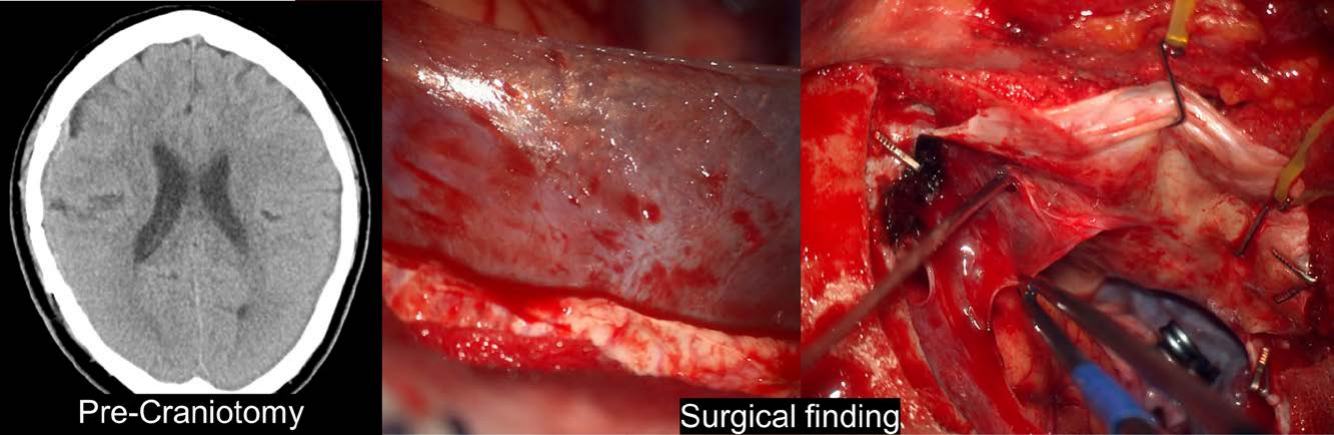
Gross findings of SDH membrane for patient 5. Brain CT showing the history of intracranial hematoma (ICH) in patient 5 and pseudomembrane seen during craniotomy were sequentially listed. Visual findings can be confirmed through an operating microscope of the pseudomembrane. CT = computed tomography, SDH = subdural hematoma.

**Figure 5.**
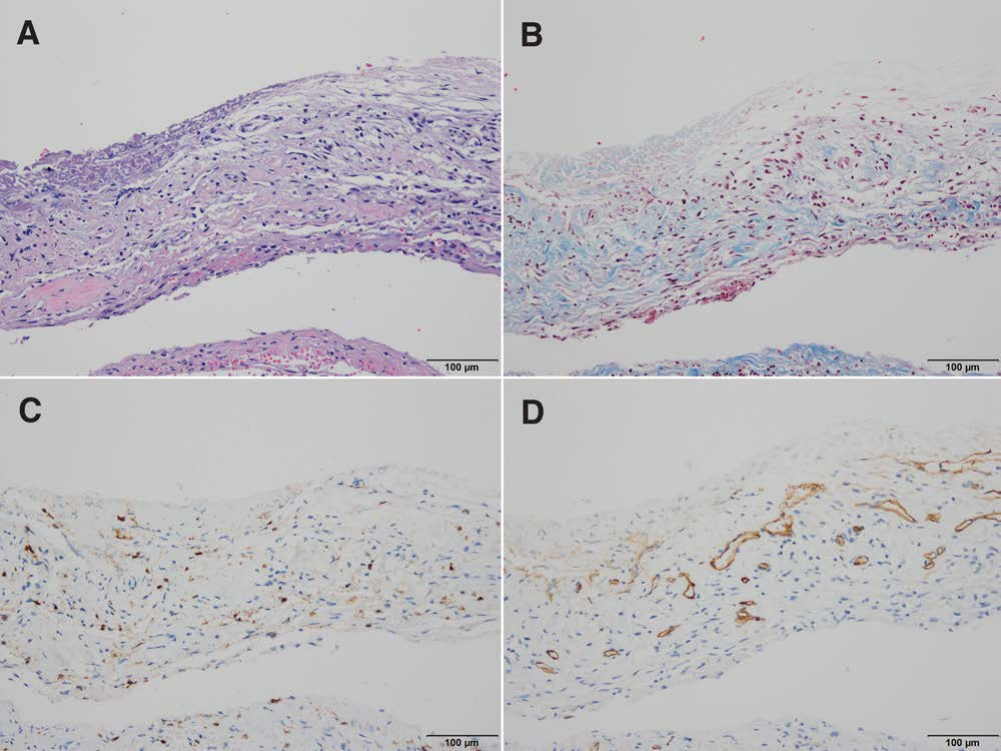
Histopathologic findings of SDH membrane for patient 5. (A) There was thin membranous structure, which is composed of compact surface layer, intermediate layer including blood vessels and inflammatory cells, and the arachnoid surface layer. B: Masson trichrome stain revealed fibrous tissue in the intermediate layer. (C&D) Immunohistochemical stain for LCA (leukocyte common antigen) and CD34 showed scattered inflammatory cells and thin walled vessels within the intermediate layer (A. Hematoxylin & eosin stain, original magnification, X200, B. Masson trichrome stain, X200, C. LCA immunohistochemical stain, X200, D. CD34 immunostain, X200). SDH = subdural hematoma.

A 72-year-old female patient was diagnosed with acute subdural hemorrhage due to head trauma but was treated conservatively without surgery. This patient underwent craniotomy aneurysm clipping 8 months after head trauma for an intracerebral aneurysm found incidentally during an outpatient visit. According to the surgical findings, there was a thin translucent membrane in the subdural space, but it was thinner and more transparent than the membrane of the first patient, and a hematoma attached to the membrane was not seen (Fig. 4). In the histopathological findings, there was a thin membranous structure composed of a compact surface layer, an intermediate layer including blood vessels and inflammatory cells, and the arachnoid surface layer on the H&E stain (Fig. 5A&B). Masson trichrome staining revealed scanty fibrous tissue in the intermediate layer. Immunohistochemical stains for LCA and CD34 showed scattered inflammatory cells and thin-walled vessels within the intermediate layer (Fig. 5C&D).

**Figure 6.**
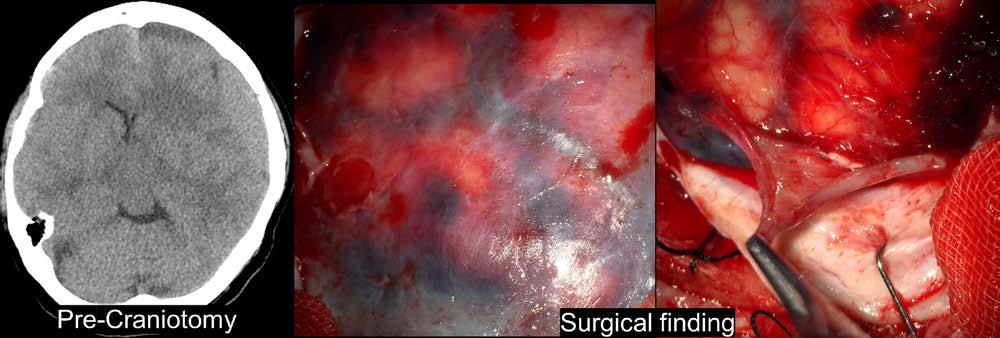
Gross findings of SDH membrane for patient 6. Brain CT showing the history of intracranial hematoma (ICH) in patient 6 and pseudomembrane seen during craniotomy were sequentially listed. Visual findings can be confirmed through an operating microscope of the pseudomembrane. CT = computed tomography, SDH = subdural hematoma.

**Figure 7.**
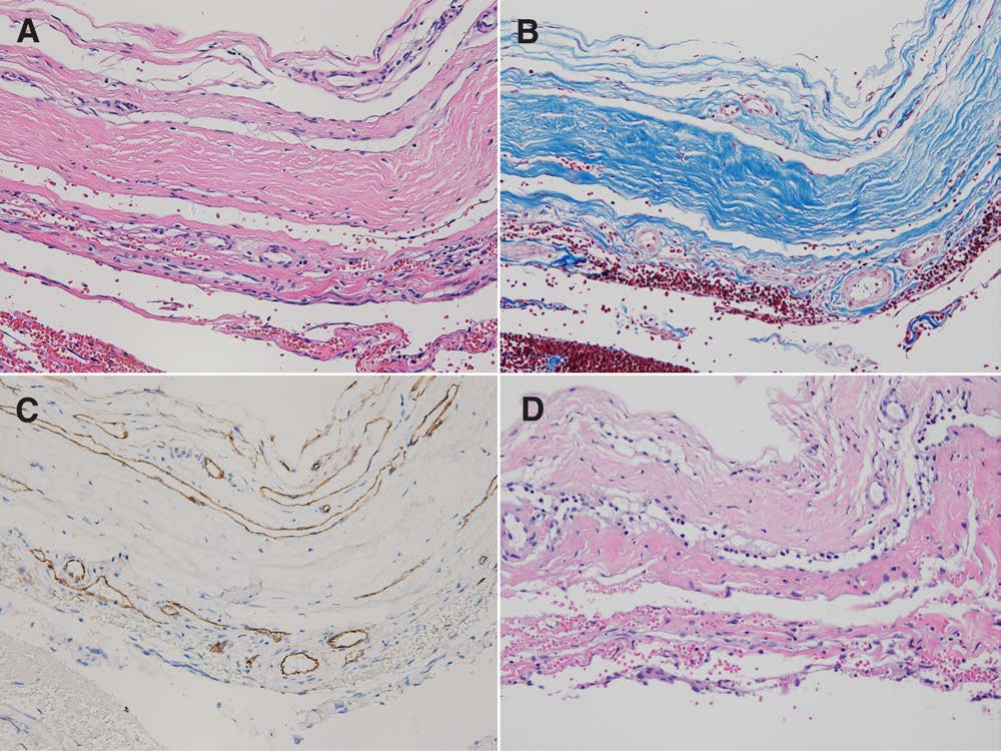
Histopathologic findings of SDH membrane for patient 6. (A) There was relatively thick membranous structure, composed of compact surface layer, intermediate layer, and the arachnoid surface layer. (B) Masson trichrome stain revealed dense collagenous tissue in intermediate layer. (C) CD34 immunohistochemical stain showed blood vessels with variable thickness. (D) Depending on the location, various degree of fibrosis was noted (A. Hematoxylin and eosin stain, X200, B. Masson trichrome stain, X200, C. CD34 immunohistochemical stain, X200, D. Hematoxylin and eosin stain, X200). SDH = subdural hematoma.

A 71-year-old female patient had a history of trephination for subdural hemorrhage 4 years ago. This patient underwent emergency craniotomy due to a traumatic parenchymal hematoma on the same side as a previous operation after a car accident. According to the surgical findings, there were traces of the previous trephination on the epidural surface. A membrane in the subdural space was less transparent and thicker than in the previous patient (Fig. 6). On the H&E stain, there was a relatively thick membranous structure composed of a compact surface layer, intermediate layer, and arachnoid surface layer (Fig. 7A&B). Depending on the location, various degrees of fibrosis were noted (Fig. 7D). Masson trichrome staining revealed dense collagenous tissue in the intermediate layer. CD34 immunohistochemical stain showed blood vessels with variable thickness (Fig. 7C).

## 3. Discussion

Although the pathophysiology of CSDH has not been clearly elucidated, many studies have been conducted on the role of the adventitia on CSDH.^[7–9]^ The pathophysiology of the adventitia on CSDH enlargement remains unclear. Currently, the self-perpetuating cycle of recurrent hemorrhage, fibrinolysis, and inflammation can lead to CSDH. The meninges are derived from the embryonic mesoderm and, as with other mesodermal tissues such as the dermis, the response to injury is the same for the initial hemorrhage and a shearing injury in the border cell layer of the meninges. In wound healing and CSDH, extravasated blood, if any, undergoes platelet activation, which causes the release of platelet-derived growth factor and clotting, which causes the release of thrombin and fibrinogen.^[10,11]^ Enlargement of CSDH is explained by recurrent hemorrhage into the subdural fluid from the outer membrane with a well-developed capillary network. Previous reports of CSDH using a scanning electron microscope did identify the outer membrane with numerous capillaries, whereas the inner membrane was thin and avascular.^[12]^ However, in our pathology report, both the inner and outer membranes showed abundant capillary networks (Figs. 3A, 5A, and 7A). In the operative microscopic view, we could see a thicker membrane covering the brain over time that could be compared more clearly using scanning electron microscope. Interestingly dense collagenous tissue was observed in the intermediate layer revealed by Masson trichrome staining. According to the results of our study, it was confirmed that dense collagenous tissue, which was initially scattered (Fig. 3B), formed a layer over time (Fig. 5B). After the layer was formed, the volume grew and became thick (Fig. 7B) and is thought to be the result of sustained wound healing. Therefore, asymptomatic recurrent hematoma was repeated.

## 4. Conclusion

This study suggests that the outer membrane of SDH is a mixture of many inflammatory cells and collagen cells in the early stages and begins to thicken after it is layered. Since the meninges, like the cutaneous dermis, are derived from the embryonic mesoderm, our hypothesis that the response to injury will also be similar to the cutaneous wound healing response is of some significance. Although the pathophysiology of CSDH has not been elucidated, we believe that our case study will provide an opportunity to better understand the inflammatory changes and angiogenesis of the SDH outer membrane.

## Acknowledgments

This study was supported by a grant from the Korea Health Technology R&D Project, Ministry of Health & Welfare, Republic of Korea (HI22C1015).

## Author contributions

**Conceptualization:** Han Seung Ryu, Sung-Pil Joo, Sung Sun Kim, Tae-Sun Kim.

**Data curation:** Han Seung Ryu, Sung-Pil Joo, Sung Sun Kim, Woo Jun Hong, Tae-Sun Kim.

**Formal analysis:** Han Seung Ryu.

**Investigation:** Han Seung Ryu, Woo Jun Hong.

**Visualization:** Han Seung Ryu, Sung Sun Kim.

**Writing – original draft:** Han Seung Ryu.

**Writing – review & editing:** Sung-Pil Joo.
